# Biomarkers on melanoma patient T Cells associated with ipilimumab treatment

**DOI:** 10.1186/1479-5876-10-146

**Published:** 2012-07-12

**Authors:** Wenshi Wang, Daohai Yu, Amod A Sarnaik, Bin Yu, Maclean Hall, Dawn Morelli, Yonghong Zhang, Xiuhua Zhao, Jeffrey S Weber

**Affiliations:** 1 Department of Cutaneous Oncology and the Donald A. Adam Comprehensive Melanoma Research Center, H. Lee Moffitt Cancer Center, Tampa, FL, USA; 2 Department of Biostatistics, H. Lee Moffitt Cancer Center, Tampa, FL, USA; 3 Department of Bioinformatics, H. Lee Moffitt Cancer Center, Tampa, FL, USA

**Keywords:** CTLA-4, Antibody, Biomarker, Melanoma

## Abstract

**Background:**

Ipilimumab induces long-lasting clinical responses in a minority of patients with metastatic melanoma. To better understand the mechanism(s) of action and to identify novel biomarkers associated with the clinical benefit and toxicity of ipilimumab, baseline characteristics and changes in CD4^+^ and CD8^+^ T cells from melanoma patients receiving ipilimumab were characterized by gene profiling and flow cytometry.

**Methods:**

Microarray analysis of flow-cytometry purified CD4^+^ and CD8^+^ T cells was employed to assess gene profiling changes induced by ipilimumab. Selected molecules were further investigated by flow cytometry on pre, 3-month and 6-month post-treatment specimens.

**Results:**

Ipilimumab up-regulated Ki67 and ICOS on CD4^+^ and CD8^+^ cells at both 3- and 6-month post ipilimumab (p ≤ 0.001), decreased CCR7 and CD25 on CD8^+^ at 3-month post ipilimumab (p ≤ 0.02), and increased Gata3 in CD4^+^ and CD8^+^ cells at 6-month post ipilimumab (p ≤ 0.001). Increased EOMES^+^CD8^+^, GranzymeB^+^EOMES^+^CD8^+^ and decreased Ki67^+^EOMES^+^CD4^+^ T cells at 6 months were significantly associated with relapse (all p ≤ 0.03). Decreased Ki67^+^CD8^+^ T cells were significantly associated with the development of irAE (p = 0.02). At baseline, low Ki67^+^EOMES^+^CD8^+^ T cells were associated with relapse (p ≤ 0.001), and low Ki67^+^EOMES^+^CD4^+^ T cells were associated with irAE (p ≤ 0.008).

**Conclusions:**

Up-regulation of proliferation and activation signals in CD4^+^ and CD8^+^ T cells were pharmacodynamic markers for ipilimumab. Ki67^+^EOMES^+^CD8^+^ and Ki67^+^EOMES^+^CD4^+^T cells at baseline merit further testing as biomarkers associated with outcome and irAEs, respectively.

## Background

T cells play a pivotal role in the development of immune tolerance to self, autoimmunity, and anti-tumor responses. Cytotoxic T Lymphocyte-Associated antigen 4 (CTLA-4) is a surface receptor on T lymphocytes that down-regulates pathways of T-cell activation [1], serving as an immune check point molecule. It is expressed intracellularly in resting T cells, and is transported to the T cell surface after activation of the T cell receptor (TCR). TCR engagement leads to tyrosine phosphorylation of CTLA-4 at position 164 (164Y) via the SRC kinase and releases it from AP50, resulting in its surface expression within 48 hours of T cell activation, leading to T cell tolerance and anergy. CTLA-4 expression is associated with decreased proliferation with cell cycle arrest at the G_1_-S interface and diminished cytokine secretion [2,3]. It decreases cell proliferation through the inhibition of mitogen-activated (MAP) kinases but promotes T cell survival through the binding of phosphoinositol-3 kinase and activating protein kinase B (PKB/AKT) resulting in T cell anergy and tolerance without the death of T cells [4]. CTLA-4 signals suppress both CD4^+^ and CD8^+^ T cell responses via a tyrosine-based inhibitory motif [5,6].

CTLA-4 blockade has antitumor activity in mice, and important effects on the breaking of tolerance [6-10]. In experiments with B16 melanoma, a therapeutic effect induced by CTLA-4 blockade with a vaccine was associated with the development of autoimmune vitiligo, suggesting that expansion of T cells recognizing melanocyte lineage antigens was associated with its therapeutic effect. Several autoimmune diseases were associated with the single nucleotide polymorphisms in the CTLA-4 gene, including hypothyroidism and type 1 diabetes [11]. In CTLA-4 ^-^/^-^ knockout mice, expansion of lymphocytes with diffuse lymphadenopathy and lymphoid infiltration of different organs occurs, consistent with a generalized expansion of T cells [12]. Similarly, both preclinical and clinical data indicate that CTLA-4 blockade results in activation and expansion of the total CD4^+^ and CD8^+^ effector T cells [13], and the breaking of self-tolerance has been shown in patients, as evidenced by the occurrence of immune-related adverse events (irAEs) observed with two different CTLA-4 antibodies, ipilimumab (Bristol Myers Squibb, Princeton, NJ) [14] and tremelimumab (Pfizer, New York, NY) [15].

Ipilimumab is a fully human CTLA-4 blocking IgG_1_ monoclonal antibody which induces long-lasting clinical responses in a minority of patients with metastatic melanoma [16-21]. Ipilimumab with or without a gp100 peptide vaccine, compared with gp100 vaccine alone, improved overall survival (OS) in patients with previously treated metastatic melanoma [22]. Ipilimumab when combined with dacarbazine improved overall survival in previously untreated patients compared to dacarbazine alone [23]. These were the first randomized Phase III trials to demonstrate a significant survival impact for patients with metastatic melanoma, yet few studies have shed light on its anti-tumor mechanism, or documented pharmacodynamic (PD) markers of the impact of ipilimumab. An increase in the absolute lymphocyte count (ALC) after 2 or 3 doses of the drug at weeks 4 and 7 has been documented [24], and may correlate with an improved outcome; increased CD4^+^ HLA-DR^+^ T cells have been shown in several studies to occur after ipilimumab therapy [25,26]; in several small cohort studies of brief duration, ipilimumab treatment increased the frequency of CD4^+^ICOS^hi^ T cells in tumors and in the circulation, and it also induced antibody reactivity against the cancer-testis antigen NY-ESO-1 [27,28]. CTLA-4 abrogating antibodies do not impact on vaccine-specific immune responses [29] and even when administered with a peptide vaccine, tumor antigen-specific responses were only modestly increased [29,30]. Recall responses to viral and other antigens were not altered by ipilimumab. In patients receiving another CTLA-4 abrogating antibody, tremelimumab, the ratio of intratumoral T cells to FoxP3 positive T regulatory cells was increased after treatment only in regressing lesions [30], suggesting a therapeutic impact of CTLA-4 abrogation on T cells infiltrating the tumor. The same investigators also demonstrated that peripheral blood Th17 cells were induced by tremelimumab [31], and that certain signaling pathways downstream of the TCR and cytokine receptor were also influenced by CTLA-4 blockade, such as increased pp38, pSTAT1 and pSTAT3, and decreased pLck, pERK1/2 and pSTAT5 levels [32]. CTLA-4 blockade also induced cell proliferation in the spleen, a secondary lymphoid organ, shown by molecular imaging with the PET probe 18 F-FLT [33]. Those investigators also reported significantly increased intratumoral CD4^+^ and mostly CD8^+^ T cell infiltration, with increased HLA-DR and CD45RO double positive cells in post tremelimumab biopsies [34] and increased expression of FoxP3.

To date, the molecular basis and mechanisms of action of ipilimumab have not been documented systematically *in vivo*. There is a critical need for biomarkers of the effects of ipilimumab as well as potential predictive markers for clinical outcome and induction of irAE. Therefore, in the current study we investigated the effects of ipilimumab on the gene expression profile of CD4^+^ and CD8^+^ T cells by microarray analysis when it was administered as adjuvant therapy to 12 patients in a trial for patients with high risk resected melanoma. We further clarified the immunophenotypic changes induced by ipilimumab on CD4^+^ and CD8^+^ T cells by a flow cytometry study of an expanded group of 37 patients and identified potential predictive biomarkers from pre-treatment specimens of 55 patients. Our studies provide evidence of candidate pre-treatment and PD biomarkers that merit further testing.

## Material and methods

### Patients

Between June 2004 and December 2008, 75 patients (39 at the University of Southern California, Norris Cancer Center and 36 at the Moffitt Cancer Center) with resected stage IIIC/IV melanoma received ipilimumab with or without a peptide vaccine. The demographic and clinic outcomes of the patients in the current study are shown in Table 1, which have been previously reported [26] with updated follow-up: three additional patients died (R DOD); one additional patient relapsed and was resected with no evidence of disease (R NED) instead of being NED. Toxicity was assessed by the National Cancer Institute Common Terminology Criteria for Adverse Events, version 3.0. The protocol was approved by the University of Southern California/Los Angeles County and University of South Florida Institutional Review Boards, and all patients provided written informed consent.

**Table 1:** **Demographics and treatment related variables of all patients in this study** (n = 55)

**Variable**	**Level**	**n (%)**
gender	F	21 (38.2)
	M	34 (61.8)
stage	IIIc	24 (43.6)
	IV	31 (56.4)
HLA A2	A2+	34 (61.8)
	A2-	21 (38.2)
dosage	10 mg/kg	40 (72.7)
	3 mg/kg	15 (27.3)
irAE	N	31 (56.4)
	Y	24 (43.6)
Outcome	NED	35 (63.6)
	Relapse	20 (36.4)

### PBMC collection, preparation and T cells purification

Apheresis with exchange of 5 to 7 liters was performed within 1 week before and 6 months after the initiation of therapy, after four doses of ipilimumab. Heparinized blood was collected after 2 doses of ipilimumab, 3 months after the initiation of therapy. PBMC were isolated from pre-, 3- and 6-month post-ipilimumab treatment specimens by Lymphoprep (Greiner Bio-One, Longwood, FL) density gradient centrifugation. PBMC were counted, then frozen in 90% heat inactivated human AB serum (HS, Omega Scientific, Tarzana, CA) and 10% dimethyl sulfoxide (DMSO) (Sigma, St Louis, MO) and stored in secured liquid nitrogen freezers at −168°C until use.

Cryopreserved pre- and 6-month post-treatment PBMC from each patient were thawed immediately into pre-warmed AIM-V media (Invitrogen Corporation, Grand Island, NY) supplemented with 5% HS. For microarray analysis, PBMCs were sorted as follows: cells were stained with CD3-FITC (BD Bioscience, San Diego, CA), CD4-PE (BD Bioscience), CD8-APC (BD Bioscience) for 30 min at 4°C. After extensive washing, PBMCs were stained with DAPI nucleic acid dye (Invitrogen) to exclude dead and dying cells. After washing with staining buffer (PBS + 0.5% HS), PBMC were re-suspended in staining buffer for flow cytometry sorting. CD3^+^CD4^+^CD8^-^, CD3^+^CD8^+^CD4^-^ T cells cell populations were collected by Aria (BD Bioscience, San Jose, CA). The purity of the sorted specific cell populations were consistently ≥ 99%.

### Microarray analysis

The sorted cells were pelleted and re-suspended in a RLT lysis buffer (Qiagen Science, Valencia, CA), and delivered to the Microarray Core (Moffitt Cancer Center, Tampa, FL) for expression array analysis. RNA from the sorted cell populations were DNase-treated and extracted using the Qiagen RNeasy mini-prep according to the manufacturer’s protocol (Qiagen). The Nugen Message Amp Premier kit was used to amplify 100 nanograms of total RNA (Nugen Technologies, San Carlos, CA). Briefly, the extracted poly-(A) RNA was specifically converted to cDNA, amplified and labeled with biotin following the procedure initially described by Van Gelder et al. [35]. Biotin-labeled cDNA was hybridized onto Affymetrix U133 Plus 2.0 microarrays. Microarray data were then analyzed using Affymetrix Expression Console. Gene detect calls were obtained using the Affymetrix MAS5 algorithm [36] to filter genes that are not expressed across the board and express value or signal intensity was calculated by the robust multi-array analysis method (RMA) developed by Irizarry et al [37]. Differential gene expressions were then assessed using Student’s t-test and false discovery rate (FDR) was estimated [38].

### Surface and intracellular staining

For cell surface staining, 1–2 × 10^6^ PBMC in 100 μl PBS were stained with the Aqua-live/dead fixable dye (Invitrogen) at 4°C for 30 min. After washing, PBMC were stained with fluorochrome-conjugated antibodies against cell surface markers prepared as a master-mix-solution for 30 min at 4°C. The antibodies for surface markers were purchased from BD Bioscience unless otherwise specified: CD3-AlexFluor 700, CD4-PECy7, CD8-PERCPCy5.5, ICOS-PE, CCR7-FITC (R&D Systems, Minneapolis, MN), CCR7-PE (R & D Systems), CXCR3-FITC (R & D Systems), CD109-PE, CD45RA-FITC, CD28-APC (eBioscience, San Diego, CA), IL-7R-APC (R & D system), CD25-FITC, TGFβR3-PE (R & D Systems) and MIC-A-APC (R & D Systems). The fluorescent-minus-one and isotype control were included in each experiment in order to appropriately set the gates. A total of 300,000 live single lymphocytes were acquired on a LSR II flow cytometer (BD Bioscience). Analysis gates were set on single live lymphocytes defined by scatter characteristics and Aqua negative staining. First, we gated lymphocytes by forward and side scatter, then gated on single cells by forward and sideward width and height. We observed single live lymphocytes by gating on aqua negatively stained single lymphocytes (Additional file 1: Figure S1 –row 1). In the whole study, we observed the biomarkers on/in CD4^+^ (Additional file 1: Figure S1-row 2, 3) and CD8^+^ T cells (Additional file 1: Figure S1-row 4, 5). The gating strategies for the biomarkers presented in this paper are shown in Additional file 1: Figure S1.

For intracellular staining, 2–3 × 10^6^ PBMC in 100 μl staining buffer were treated as above, then fixed with a freshly prepared Fixation/Permeabilization working solution (eBioscience) at 4°C for 30 min. After wash with permeabilization buffer (eBioscience), the cells were stained with intracellular markers: Ki67-FITC (Abcam, Cambridge, MA), EOMESodermin (EOMES)-Alex Fluor 647 (eBioscience), granzyme B-FITC, perforin-PE, FoxP3 –APC (eBioscience), GATA 3-Alex Fluor 647 (eBioscience), cleaved caspase III-PE for 30 min at 4°C. Cells were then washed in the permeabilization buffer and resuspended in staining buffer for flow cytometry acquistion.

For the standardization of flow cytometry assays and the consistency of daily performance, a normal PBMC sample was stained and run on flow cytometry in parallel with the subject samples and Spherotech rainbow fluorescent particles (Spherotech, Inc, Lake Forest, IL) were run before running samples for alignment of the optical system of the flow cytometer in each channel.

### Flow cytometry analysis

Flow cytometry data were analyzed using Flowjo software (Version 9.0.2, Tree Star, Inc., Asland, OR). The mean fluorescence intensity (MFI) and percent of positive expression (%) of each marker were measured for CD4^+^ and CD8^+^ T cells.

### Statistical analysis

Descriptive summary statistics, e.g., frequency and % for discrete variables and mean (standard deviation: SD), interquartiles and median (range) for continuous variables, were reported as appropriate. Both absolute change and fold change, i.e., post-pre and (post-pre)/pre, were analyzed to account for a potentially better normalization under the log scale for the immunological biomarkers under investigation. Median changes and interquartiles were reported for the biomarker change variables due to concerns on sometimes small sample sizes and the underlying normality assumption required for a valid confidence interval. Wilcoxon signed-rank test was used to test whether changes in immunological biomarker expression levels between pre-, to 3- and 6-month post-ipilimumab were statistically significant. Both point estimates and their confidence intervals (CIs) were provided for parameters of interest, e.g., odds ratio (OR). Confidence intervals were set at the 95% level. Univariate logistic regression models were employed to explore the effects of baseline and absolute change as well as fold change of each biomarker on disease outcome (Relapsed vs. NED) and irAE (Yes vs. No). The CI for OR based on the univariate logistic regression model results was calculated for an increment that equals to one half of the interquartile range of the corresponding change variable for each of the biomarkers. Baseline biomarkers were also dichotomized using their respective medians and then correlated with the disease outcome (Relapsed vs. NED) and irAE (Yes vs. No) using the 2x2 contingency table approach. The resulting ORs, their CIs and Fisher test p-values were reported. The Kaplan-Meier product-limit analysis and log-rank test were employed to address the question of whether relapse-free survival was associated with any of the dichotomized biomarkers at baseline. A p-value of ≤ 0.05 was considered statistically significant and no multiple comparison adjustments were made in this exploratory biomarker study except for the gene microarray part of the data analyses. All statistical analyses were performed using SAS version 9.

## Results

### Gene expression of CD4^+^ and CD8^+^ T cells induced by ipilimumab

To assess the effect of ipilimumab on overall gene expression in CD4^+^ and CD8^+^ T cells *in vivo,* microarray analysis was conducted on T cells purified by flow cytometry from PBMCs collected from 12 resected melanoma patients pre- and 6-month post-ipilimumab treatment (Additional file 1: Table S1). Differentially expressed genes were selected with a paired student T-test p-value of 0.03 or less (p ≤ 0.03) and a fold change of the group mean of 1.2 or greater (≥ 1.2) or 0.8 or less (≤ 0.8). Table 2a and 2b show some immune-related genes from the top differentially expressed genes in CD4^+^ and CD8^+^ T cells, respectively, after ipilimumab treatment. The genes were categorized by biological process and/or gene function: cell cycle, cytokine-related, chemokine-related, T cell activation and proliferation signal-related, survival and apoptotic signal-related, kinase-related and heat shock protein family-related genes. It is worth noting that the genes most impacted by ipilimumab were cell cycle related in both CD4^+^ and CD8^+^ T cells. Ipilimumab appeared to release the arrested cell cycle in CD4^+^ and CD8^+^ T cells as evidenced by highly significant and consistent up-regulation of CDC2 (Table 2a and 2b), and other cell cycle related genes (data not shown).

**Table 2:** **Microarray data for immune related genes from CD4**^**+ **^**T cells (Table 2a) and CD8**^**+ **^**T cells (Table 2b) with fold increases over baseline control samples, p-values for the differences indicated, FDR for the false discovery rate, and the mean ± SD of pre and post samples for the gene expression ranges**

**Gene symbol**	**Gene name**	**Fold-change**	**p-value**	**FDR**	**Pre-Mean ±SD**	**Post-Mean** ±**SD**
a. Genes impacted by ipilimumab in CD4+ T cells						
**Cell cycle related**						
CDC2	cell division cycle 2, G1 to S and G2 to M	1.98	0.009	0.0409	18.61 ± 4.41	36.92 ± 21.36
		2.78	0.0142	0.0432	66.68 ± 59.31	185.59 ± 185.36
TYMS	thymidylate synthetase	2.77	0.0075	0.0408	129.75 ± 58.10	358.99 ± 254.47
CCNB2	cyclin B2	2.08	0.0142	0.0434	28.17 ± 11.75	58.49 ± 45.13
CDK7	cyclin-dependent kinase 7	1.26	0.0074	0.0408	214.08 ± 75.01	270.13 ± 106.34
**Cytokines-related**						
PGDS	prostaglandin D2 synthase, hematopoietic	2.2	0.0024	0.0393	14.20 ± 5.42	31.30 ± 14.57
		1.51	0.0048	0.04044	489.68 ± 470.87	740.39 ± 581.83
TGFBR3	transforming growth factor β RIII	1.39	0.0111	0.0411	505.71 ± 304.47	701.22 ± 246.45
IFNy	interferon, γ	1.46	0.0255	0.0751	138.35 ± 104.72	201.55 ± 120.78
IL-7	interleukin 7	1.28	0.0169	0.0511	15.43 ± 10.42	19.73 ± 8.89
STAT1	signal transducer and activator of transcription 1	1.28	0.0081	0.0409	1141.96 ± 330.11	1460.67 ± 577.64
SCYE1	small inducible cytokine subfamily E, member 1	1.27	0.0103	0.041	278.24 ± 100.74	352.82 ± 149.81
		0.80	0.0061	0.0406	5219.38 ± 1457.55	4291.38 ± 1772.32
IL-7R	interleukin 7 receptor	0.74	0.0011	0.0375	3309.89 ± 1296.32	2467.81 ± 1094.22
IL-2Rα	interleukin 2 receptor, α	0.69	0.01811	0.0544	113.56 ± 58.89	79.12 ± 39.28
IL-15	interleukin 15	0.74	0.0032	0.0398	54.76 ± 39.59	40.63 ± 31.09
TNFSF8	tumor necrosis factor superfamily, member 8	0.73	0.0202	0.0605	248.08 ± 148.25	181.53 ± 92.03
**Chemokines-related**						
ITGB1	integrin, β1 (antigen CD29)	1.29	0.0055	0.0405	1449.91 ± 772.02	1867.82 ± 802.10
		1.20	0.0012	0.0375	396.68 ± 336.59	476.25 ± 376.51
CXCR3	chemokine receptor 3	1.29	0.0286	0.0825	47.04 ± 23.35	60.53 ± 33.18
CXCR7	chemokine receptor 7	0.75	0.0205	0.0614	83.62 ± 25.83	63.45 ± 25.78
ITGA6	integrin, α6	0.72	0.0211	0.063	1166.8 ± 1046.57	837.4 ± 796.19
**Activation, proliferation and differentiation-related**						
MKI67	antigen identified by monoclonal antibody Ki-67	1.98	0.0165	0.0498	41.58 ± 22.80	82.16 ± 62.67
		1.69	0.0104	0.0410	19.37 ± 9.50	32.73 ± 20.37
ICOS	inducible T-cell costimulator	1.49	0.0011	0.0375	587.76 ± 294.69	874.26 ± 397.84
GATA3	GATA binding protein 3	1.43	0.0124	0.042	284.77 ± 189.82	406.03 ± 268.34
		1.37	0.0023	0.0392	163.55 ± 83.74	224.53 ± 114.73
CTLA-4	cytotoxic T-lymphocyte-associated protein 4	1.37	0.0249	0.0734	511.58 ± 260.28	700.23 ± 359.54
MICA	MHC class I polypeptide-related sequence A	0.7	0.0015	0.0379	120.33 ± 67.61	84.36 ± 51.72
**BCL family-related**						
Bcl3	B-cell CLL/lymphoma 3	1.56	0.0036	0.0400	199.55 ± 112.65	311.36 ± 120.66
BAK1	Bcl2-antagonist/killer 1	1.26	2.00E-04	0.0365	130.50 ± 27.61	164.92 ± 33.45
		1.24	0.0067	0.0407	314.36 ± 77.54	388.54 ± 79.60
Bcl2L11	Bcl2-like 11	1.21	0.0369	0.1053	442.48 ± 299.18	536.88 ± 388.57
BCLAF1	Bcl2-associated transcription factor 1	1.21	0.0234	0.0695	61.14 ± 14.91	73.89 ± 26.64
		0.72	0.0158	0.0479	189.15 ± 81.00	137.91 ± 40.11
Bcl2	B-cell CLL/lymphoma 2	0.63	0.0050	0.0404	427.68 ± 327.43	272.97 ± 207.62
**Apoptosis, MAP kinase and protein kinase-related**						
ANXA5	annexin 5	1.32	0.0271	0.0794	599.68 ± 269.08	793.89 ± 425.06
MAPK6	mitogen-activated protein kinase 6	1.31	0.0261	0.0768	209.49 ± 142.30	273.63 ± 202.36
PPP1CC	protein phosphatase 1, catalytic subunit, γ isoform	1.23	0.0152	0.0462	998.56 ± 432.59	1225.87 ± 571.01
CASP7	caspase 7, apoptosis-related cysteine peptidase	1.22	0.0145	0.0441	141.11 ± 41.72	171.83 ± 48.77
MAP2K6	mitogen-activated protein kinase kinase kinase 6	0.77	0.0105	0.0410	77.3 ± 41.24	59.77 ± 33.17
ANXA11	annexin 11	0.75	0.0003	0.0368	93.23 ± 20.45	70.21 ± 31.98
ATF7	activating transcription factor 7	0.75	0.0252	0.0743	170.44 ± 120.64	128.30 ± 107.21
MAP4K4	mitogen-activated protein kinase kinase kinase 4	0.63	0.0095	0.041	137.49 ± 51.60	86.97 ± 29.96
b. Genes impacted by ipilimumab in CD8^+^ T cells						
**Cell cycle-related**						
CDC2	cell division cycle 2, G1 to S and G2 to M	1.81	0.03	0.0897	18.37 ± 6.09	33.23 ± 21.15
CDCA7	Cell division cycle associated 7	1.67	0.0113	0.0647	87.63 ± 93.37	146.50 ± 111.23
**Cytokines-related**						
TNFSF4	tumor necrosis factor superfamily, member 4	0.58	0.0082	0.0647	40.88 ± 27.10	23.93 ± 14.71
**Chemokines-related**						
ITGAV	integrin, αV (antigen CD51)	0.80	5.00E-04	0.0647	114.65 ± 54.10	91.78 ± 42.91
**Activation, Differentiation and Interaction-related**						
HLA-DRB4	major histocompatibility complex, class II, DR β4	1.4	0.0163	0.0647	28.04 ± 26.29	39.18 ± 37.40
		1.39	0.0100	0.0647	178.84 ± 104.07	248.93 ± 148.36
GATA3	GATA binding protein 3	1.35	0.0026	0.0647	139.84 ± 44.14	188.83 ± 75.51
HLA-DRA	major histocompatibility complex, class II, DR α	1.38	0.0193	0.0647	540.53 ± 402.57	745.47 ± 350.32
CD6	CD6 molecule (CD166 receptor)	1.32	0.0248	0.0754	179.47 ± 95.79	236.58 ± 157.76
CD5	CD5 molecule	1.22	0.0181	0.0647	103.58 ± 38.21	126.47 ± 45.17
EOMES	Eomesodermin	0.69	0.0292	0.0877	1063.26 ± 642.42	738.05 ± 445.38
**Apoptosis, MAP Kinases-related**						
TIAM1	T-cell lymphoma invasion and metastasis 1	1.68	0.0105	0.0647	83.63 ± 67.00	140.61 ± 116.68
ANXA5	annexin 5	1.31	0.0114	0.0647	576.93 ± 278.33	747.53 ± 316.78
API5	apoptosis inhibitor 5	1.24	0.0088	0.0647	187.92 ± 118.48	233.04 ± 151.93

Increases of greater than 1.2 fold and decreases of 0.8 fold or less are shown. Multiple values refer to different expressed sequence tags from the same gene.

### Pharmacodynamic effects of ipilimumab on T cells

To verify changes in selected molecules from the microarray analysis and further investigate the mechanism of action of ipilimumab, a flow cytometry study was undertaken with the pre-, 3-month and 6-month post-ipilimumab PBMCs from expanded groups of 55, 25 and 37 patients, respectively (Table 2, Additional file 1: Table S1b and S1c), overlapping the microarray cohort of 12 but limited to all patients with sufficient PBMC available for analysis. In addition to the selected immunological biomarkers, we also measured CD4^+^ and CD8^+^ T cell effector-memory/naive phenotypes. We observed that naïve CD4^+^ and CD8^+^ T cells (CCR7^+^CD45RA^+^) were significantly reduced 6 months after ipilimumab, with a corresponding significant increase in CD8^+^ central memory and CD4^+^ effector memory T cells (data not shown). We report below biomarkers that significantly changed both in absolute value and by fold change for % (positivity) with p ≤ 0.02 by Wilcoxon.

### **Increased Ki67 expression in, and ICOS on CD4**^+^**and CD8**^+^**T cells 3 and 6 months after ipilimumab**

The Ki-67 protein (also known as MKI67) is a cellular marker for proliferation [39] and cell cycling [40] and is an indicator of the growth fraction of a given cell population. As demonstrated in Table 3a and Table 3b, the % of Ki67^+^/CD4^+^ and Ki67^+^/CD8^+^ T cells were significantly increased in both 3-month and 6-month post-treatment PBMCs with p ≤ 0.0009 for all by Wilcoxon test and a median fold-increase ranging from 0.54 to 1.50. The increase in Ki67 ^+^/CD4^+^ T cells was higher at 3-month post ipilimumab treatment than at 6-month post treatment, but the increase of Ki67^+^/CD8^+^ T cells was higher at 6-month post ipilimumab than at 3-month post ipilimumab.

**Table 3:** Flow cytometry data for different phenotypic biomarkers from CD4 or CD8 T cells with absolute changes and fold-changes over baseline control samples at 3- (Table 3a) and 6-month (Table 3b) post ipilimumab with p-values for the differences shown

**Biomarker**	**n**	Median change * (Q1, Q3)	**Wilcoxon p-value**	Median foldchange * (Q1, Q3)	**Wilcoxon p-value**
a. Statistical analysis of changes in biomarkers at 3 months					
%-ICOS-CD4	25	13.00 (1.15, 20.69)	<.0001	1.35 (0.23, 3.62)	<.0001
%-ICOS-CD8	25	4.19 (2.07, 7.75)	<.0001	2.90 (1.25, 6.72)	<.0001
%-Ki67-CD4	24	3.62 (0.74, 6.94)	<.0001	1.50 (0.18, 2.64)	<.0001
%-Ki67-CD8	24	1.49 (−0.10, 4.86)	0.0009	0.54 (−0.03, 2.20)	0.0003
%-CCR7-CD8	25	−3.98 (−11.40, 1.30)	0.0122	−0.14 (−0.27, 0.04)	0.018
%-CD25-CD8	25	−1.60 (−5.68, -0.70)	<.0001	−0.42 (−0.56, -0.25)	<.0001
b. Statistical analysis of changes in biomarkers at 6 months					
%-ICOS-CD4	37	9.45 (3.13, 14.57)	<.0001	1.66 (0.70, 2.42)	<.0001
%-ICOS-CD8	37	3.07 (1.62, 5.36)	<.0001	1.34 (0.63, 3.30)	<.0001
%-Ki67-CD4	35	2.33 (1.37, 6.97)	<.0001	0.87 (0.29, 2.12)	<.0001
%-Ki67-CD8	36	1.88 (0.13, 4.91)	0.0004	0.55 (0.04, 1.52)	<.0001
%-Gata3-CD4	28	3.76 (0.28, 8.04)	0.0004	0.94 (−0.01, 1.62)	<.0001
%-Gata3-CD8	28	2.07 (0.59, 4.02)	0.0006	0.59 (0.09, 1.23)	<.0001
%-CCR7-CD4	37	−2.90 (−6.80, -0.40)	0.0017	−0.03 (−0.09, -0.01)	0.006

*(Q1, Q3) are first and third interquartiles.

ICOS is a T cell surface molecule structurally related to CD28 and CTLA-4 [41]. It is expressed at low levels on resting naïve T cells and is rapidly up-regulated following TCR ligation and CD28 costimulation [42]. After ipilimumab treatment, ICOS was increased significantly on CD4^+^ and CD8^+^ T cells in both 3-month and 6-month post-treatment PBMCs (p ≤ 0.0001 with a median fold-increase of 1.34 to 2.90), with higher increases on CD4^+^ than on CD8^+^ T cells and higher absolute increases at 3-month than at 6-month post-treatment (Table 3a and 3b).

### **Decreased CCR7 on CD8**^**+**^**and CD4**^+^**T cells after ipilimumab**

The % of CCR7^+^/CD8^+^ T cells was decreased at 3-month post-ipilimumab (p ≤ 0.018 with a median fold-decrease of 0.14, Table 3a). Similarly, the % of CCR7^+^/CD4^+^ T cells was down-regulated at 6-month post-ipilimumab treatment (p ≤ 0.006 with a median fold-decrease of 0.03, Table 3b).

### Decreased CD25 on CD8^+^ after ipilimumab

CD25 is a T cell activation marker, the % of CD25 expression on CD8^+^ T cells were decreased at 3-month post-ipilimumab (p ≤ 0.0001 with a median fold-decrease of 0.42, Table 3a).

### **Increased GATA3 expression in CD4**^+^**and CD8**^+^**T cells**

GATA3 is a transcription factor that is a marker for Th2 polarization and is associated with the generation of Th2 cytokines IL-4, IL-5 and IL-10. After treatment with ipilimumab, GATA3 expression was increased significantly in CD4^+^ and CD8^+^ T cells at 6-month post-ipilimumab treatment (p ≤ 0.0006 with a median fold-increase of 0.94 and 0.59, Table 3b).

### Surrogate biomarkers on/in T cells associated with relapse or irAE

In an univariable logistic regression analysis using changes in expression by flow cytometry as a continuous variable one at a time, only absolute decreases in % of Ki67^+^EOMES^+^CD4^+^ and % of CCR7^+^CD8^+^, and increases in % of EOMES^+^CD8^+^ and % of GranzymeB^+^ EOMES^+^CD8^+^ T cells were associated with a higher odds of relapse with p < 0.04 (OR for increment of half the interquartile range of the corresponding change ranging from 0.17 to 0.50 for the negative association and from 2.63 to 5.00 for the positive association, Table 4a). Only absolute decrease in % of Ki67^+^CD8^+^ T cells was associated with a development of irAE with p = 0.02 (OR = 0.47, Table 4b).

**Table 4:** Association of phenotypic changes on T cells with clinical outcome (Relapse vs. NED) (Table 4a) and irAE (Yes vs. No) (Table 4b) was based on univariable logistic regression modeling

**Biomarker**	**n**	**Slope**	**p-value**	**Â½ IQR**	**OR (95% CI)**
a. Association of changes in biomarkers at 6 months with outcome (Relapse vs. NED)					
%-Ki67 + EOMES + CD4+	19	−3.5232	0.0285	0.5	0.17 ( 0.04, 0.83)
%-CCR7 + CD8+	37	−0.0947	0.0379	7.4	0.50 ( 0.26 0.96)
%-EOMES + CD8+	36	0.1758	0.0072	5.5	2.63 ( 1.30, 5.32)
% EOMES + GranzymeB + CD8+	19	0.2774	0.0293	5.8	5.00 ( 1.18, 21.23)
b. Association of changes in biomarkers at 6 months with irAE (Yes vs. No)					
%-Ki67 + CD8+	36	−0.3167	0.0217	2.4	0.47 ( 0.24, 0.89)

A total of 37 paired specimens of PBMC prior to and after four doses of ipilimumab were analyzed by flow cytometry for the indicated markers. CCR7 was stained extracellularly, EOMES, granzyme B and Ki67 were stained intracellularly per Materials and Methods.

**Table 5:** a, Association between outcome (Relapse vs. NED) and dichotomized biomarkers at baseline. b, Association of baseline dichotomized biomarkers with irAE (Yes vs. No) for a total 55 patients

**Biomarker**	**Outcome**	**n (%)**	**Odds Ratio**	**Fisher**
			**(95% CI)**	**p-value**
a. Association between outcome (Relapse/NED) and dichotomized baseline biomarkers by median				
%-Ki67 + EOMES + CD8+ (n = 39)			11.25 ( 2.52, 50.27)	0.0012
<=2.11	Relapse	15 ( 75.0)		
<=2.11	NED	5 ( 25.0)		
2.11	Relapse	4 ( 21.1)		
>2.11	NED	15 ( 78.9)		
%-EOMES + CD8+ (n = 54)			3.77 ( 1.16, 12.27)	0.0473
<=55.6	Relapse	14 ( 51.9)		
<=55.6	NED	13 ( 48.1)		
>55.6	Relapse	6 ( 22.2)		
>55.6	NED	21 ( 77.8)		
b. Association between irAE (Yes/No) and dichotomized baseline biomarkers by median				
%-Ki67 + EOMES + CD4+ (n = 39)			8.00 ( 1.74, 36.70)	0.0079
<=0.446	Yes	12 ( 60.0)		
<=0.446	No	8 ( 40.0)		
>0.446	Yes	3 ( 15.8)		
>0.446	No	16 ( 84.2)		

Ki67 and EOMES that was stained intracellularly in a total of 36 paired specimens of PBMC prior to and after four doses of ipilimumab was analyzed by flow cytometry.

### Pre-treatment biomarkers on/in T cells associated with outcome and irAE

An analysis of biomarkers at baseline dichotomized by their medians revealed that a low % of Ki67^+^EOMES^+^CD8^+^ T cells, and a low % of EOMES^+^CD8^+^ T cells were significantly associated with relapse (p =0.001, and 0.047 with OR = 11.25, and 3.77, respectively; Table 5a). These pre-treatment biomarkers were also confirmed in a univariate logistic regression analysis to be associated with relapse (data not shown). A similar analysis of dichotomized baseline biomarkers by their medians showed that a low % of Ki67^+^EOMES^+^CD4^+^ T cells was associated with occurrence of irAE (p =0.008 with OR = 8.00, Table 5b).

### Pre-treatment biomarkers on/in T cells associated with RFS

**Figure 1:**
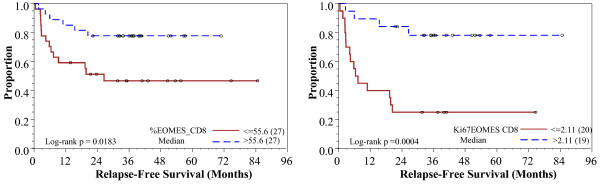
**a: Kaplan-Meier relapse-free survival curves comparing patients with (high) greater than median baseline % of EOMES**^**+**^**CD8**^**+ **^**to patients with (low) less than median % of EOMES**^**+ **^**CD8**^**+**^**. b**, Kaplan-Meier relapse-free survival curves comparing patients with high baseline % of Ki67^+ ^EOMES^+^/CD8^+^ to patients with low % of Ki67^+^EOMES^+^/CD8^+^.

Our analysis highlighted the potential importance of EOMES, a transcription factor in the T-box family and involved in the regulation of INF-γ, granzyme B and perforin production by CD8^+^ T cells [43]. To better understand the potential role of EOMES^+^CD8^+^ T cells in ipilimumab treatment, we stratified pre-treatment specimens by the median % of EOMES^+^CD8^+^ T cells. Patients with higher baseline % of EOMES^+^CD8^+^ T cells had a significantly improved relapse-free survival (RFS) compared to those with a lower basal level of EOMES^+^CD8^+^ T cells (p = 0.02 by log-rank test, Figure 1a). The patients were also stratified by the median % of Ki67^+^EOMES^+^CD8^+^ T cells. Patients with a higher proportion of Ki67^+^EOMES^+^CD8^+^ T cells had significantly improved RFS compared with those with a lower % of Ki67^+^EOMES^+^CD8^+^ T cells (p = 0.0004, Figure 1b).

We also assessed these biomarkers in 11 pheresis specimens from normal donors as a reference. Levels of the most significant biomarker-% of EOMES^+^Ki67^+^CD8^+^ T cells from the 11 normals and the 55 melanoma patients in this trial are presented in Additional file 1: Figure S2. The median % of EOMES^+^Ki67^+^CD8^+^ T cells for the 11 normals was 1.57, very similar to the median of 1.50 for the relapsed patients (Wilcoxon Rank-sum test p = 0.38). For the NED group, the median % of EOMES^+^Ki67^+^CD8^+^ T cells was 2.50, significantly higher than that of relapsed patients (p = 0.003) as well as that of the normal patient specimen (p = 0.026).

## Discussion

In the current study, we utilized an expression microarray analysis of flow cytometry-purified CD4^+^ and CD8^+^ T cells to define immunologically important gene products expressed by T cells from patients with high-risk melanoma that were significantly altered after ipilimumab. We then investigated selected T cell markers by performing flow cytometry analysis of membrane-bound and intra-cellular determinants using a larger group of samples, including 25 sets of pre-, 3-month, and 37 sets of pre- and 6-month post-treatment PBMCs and 55 pre-treatment PBMC samples. We aimed to define potential biomarkers for ipilimumab treatment and determine if they were associated with clinical outcome and induction of irAE. The patients’ age, gender, disease stage and the dosage of ipilimumab (3 vs. 10 mg/kg) were not significantly associated with either outcome or irAE, nor did they significantly impact on the studied biomarkers (data not shown).

Previous investigations have focused on observations in peripheral blood or tumors from patients who have received CTLA-4 antibodies. CTLA-4 abrogation is expected to result in diffuse CD4^+^ and CD8^+^ T cell expansion and activation, based on the extensive lymphoid hypertrophy seen in the CTLA-4−/− knockout mice, and on the rise in ALC commonly observed in patients receiving ipilimumab. Several investigators described increases in CD4^+^HLA-DR^+^ activated T cells, reductions in naïve T cells and an increase in central memory cells in those patients. In the current study, we also observed similar changes (data not shown). Augmented Th17 cells and increases in CD4^+^ICOS^+^ cells have also been described after CTLA-4 blockade, however no detailed analysis has explored the effects of CTLA-4 blocking antibodies on the expression of different immune markers on T cells.

In the current work, a number of biomarkers on and in T cells were significantly elevated after repeated injections of ipilimumab. Increased ICOS on CD4^+^ and CD8^+^ T cells was a pharmacodynamic marker for ipilimumab treatment, confirming results from prior studies. Increased CD4^+^ICOS^Hi^ T cells have been reported to infiltrate tumor tissues and are found in peripheral blood after ipilimumab. In a small number of patients, a significant increase of CD4^+^ICOS^Hi^ and CD8^+^ICOS^Hi^ T cells was observed at weeks 3 and 7. By week 24, CD4^+^ICOS^Hi^ T cells had returned to baseline values. Increased ICOS on CD4^+^ T cells was more pronounced after treatment with ipilimumab at 10 mg/kg/dose, with concomitant increases in CD8^+^ICOS^Hi^ T cells which were not observed after treatment with the 3 mg/kg/dose of antibody. In that small study, a sustained increase in frequency of CD4^+^ICOS^Hi^ T cells was associated with overall survival at week 24 [27]. Our current study is consistent with a significant increase of ICOS expression on both CD4^+^ and CD8^+^ T cells not only at 3- but also at 6- months (24 weeks) post ipilimumab independent of age, sex, metastatic stage of disease, or dose of ipilimumab. Changes in ICOS expression on CD4 or CD8 T cells were not associated with clinical outcome in our analysis. ICOS is not necessary for Th17 differentiation, but it is required for the expansion of the Th17 compartment [44]. ICOS expressing cells may also demonstrate anti-tumor reactivity and be responsible in part for the anti-tumor effects of ipilimumab [45].

We observed that ipilimumab resulted in consistently elevated Ki-67, a nuclear proliferation marker, in both CD4^+^ and CD8^+^ T cells 3- and 6- month post ipilimumab. Interestingly, there was no alteration observed in the frequency of Ki67 positive cells among tumor infiltrating lymphocytes when post –tremelimumab biopsies were compared to baseline biopsies [34]. The reason for increased Ki-67 in CD4^+^ and CD8^+^ T cells in peripheral blood, but not in the tumor site might be due to the differences in the CTLA-4 blocking antibodies or the different time points for sample harvesting, or lymphocytes may proliferate in lymphoid organs [33] and the peripheral blood compartment before they infiltrate into tumor sites.

The activation marker ICOS, proliferation marker Ki67, and the Th2 polarizing transcription factor GATA3 were also elevated on CD4^+^ and CD8^+^ T cells, suggesting that prolonged CTLA-4 blockade induced the generalized expansion of activated T cells that might generate Th2 cytokines IL-4, IL-5 and IL-10. Elevated levels of T cell IFN-γ were detected on the microarray analysis, in contradistinction to the increase in GATA3. The observation that CCR7 was decreased on CD8^+^ and CD4^+^ T cells was consistent with the diminution of naïve T cells in the circulation.

Novel findings from the current study were that a variety of immune markers either rose or declined on CD4^+^ and CD8^+^ T cells after treatment with ipilimumab. The microarray data defined a larger group of altered genes that increased by at least 1.2 fold or decreased by 0.8 fold in the CD4^+^ cells than in CD8^+^ T cells, suggesting that effects of CTLA-4 blockade with ipilimumab were predominantly on the CD4^+^ T cell population. CTLA-4 signals block the lymphocyte cell cycle at the G1 to S transition. Ipilimumab released the cell cycle arrest of CD4^+^ and CD8^+^ T cells, as demonstrated by the significant alteration of a group of cell cycle genes (data not shown).

Our data are consistent with other studies showing that the effects of CTLA-4 blockade are more prominent on CD4^+^ T cells, and that T lymphocytes are broadly expanded. Our data on the association of relapse with phenotypic changes on CD8^+^ T cells are novel and point to the importance of this cell population in the mechanism of ipilimumab’s anti-tumor effect. The use of microarrays and T cell phenotypic analysis to confirm the changes in gene expression would not have detected the alterations seen in detailed phosphoflow assays by Comin-Anduix et al [32]. Interestingly, the microarray analysis in the current work did not show that major alterations were seen in downstream TCR and cytokine signaling molecules, so we cannot confirm or refute their data. We did not see significant changes in FoxP3 or GITR expression, consistent with our own and others’ work, nor were cytokine levels appreciably altered other than gamma interferon. While we focused on the effects of ipilimumab on T cells as an effective immunotherapeutic agent, it might also have a wider impact on the immune system, not only on CD4^+^ and CD8^+^ T cells as in our current study, but also on CD14^+^ monocytes [32] and B cells [46].

Ipilimumab may also rescue tumor-impaired IFN-γ pathways in CD4^+^ T cells [47], since a number of genes associated with IFN-γ signals such as STAT1, ISG15, GBp1, and EIF2AK2 were up-regulated by ipilimumab (data not shown). Up-regulation of STAT1 has been confirmed by phosphoflow [32] after tremelimumab.

Eomesodermin (EOMES) is an important transcription factor controlling the function of effector CD8^+^ T cells [43]. The expression of EOMES in CD8^+^ T cells may reflect a critical basal level of immune competence of the melanoma patient. CD8^+^ T cells play a vital role in the immune response to melanoma, and their baseline level of activation and function may be a *sine qua non* for the therapeutic effect of ipilimumab. CTLA-4 signaling may specifically target EOMES resulting in reduced IFN-γ and granzyme B expression by selectively inhibiting accumulation of EOMES mRNA and protein [48]. Ectopic expression of EOMES reversed CTLA-4 mediated inhibition of effector molecules, and CTLA-4^-^/ CD8^+^ T cells had greatly enhanced IFN-γ and granzyme B production, as well as enhanced cytolytic function and increased expression of EOMES [48]. The increase in IFN-γ signals and up-regulated % of granzyme B expression on EOMES^+^/CD8^+^ T cells (p = 0.003, data not shown) in our current study would suggest ipilimumab interferes with the down-regulation of EOMES and its downstream signals, IFN-γ and granzyme B, that is mediated by CTLA-4 engagement. The pre-treatment expression of EOMES in CD8^+^ T cells and the level of Ki67^+^EOMES^+^CD8^+^ T cells are indices that appear to be associated with decreased risk of relapse and prolonged RFS after adjuvant treatment with ipilimumab, and these biomarkers could potentially be predictive for its benefit.

How do these data inform us about the effects of CTLA-4 abrogation in patients? First, the pharmacodynamic effects of ipilimumab *in vivo* can help clarify its mechanism(s) of action. Next, some of the observed effects may be surrogate biomarkers for either toxicity or benefit, allowing an early prediction of those phenomena in patients. Some biomarkers on CD8^+^ T cells were associated with relapse in this study, and that result needs to be validated in a larger prospective study of patients with metastatic, unresectable melanoma. Our microarray data also indicated that other genes whose expression is altered in CD4 and CD8 T cells after ipilimumab might be worth exploring. Finally, some of the biomarkers that we defined herein may be predictive for the effects of ipilimumab in patients, allowing better patient selection, or may simply be prognostic for outcome independent of specific treatment. We showed that EOMES^+^CD8^+^ T cells and Ki67^+^EOMES^+^CD8^+^ T cells are biomarkers associated with clinical outcome with ipilimumab. Further studies to validate these observations prospectively may define clinically useful predictive biomarkers to select patients for ipilimumab treatment.

## Abbreviations

CTLA-4: Cytotoxic T Lymphocyte-Associated antigen 4; PBMC: Peripheral Blood Mononuclear Cell; HS: heat inactivated human serum; TCR: T cell receptor; FoxP3: Forkhead box protein P3; EOMES: Eomesodermin; FDR: False discovery rate; NED: No evidence of disease; IrAEs: immune-related adverse events; RFS: relapse-free survival.

## Competing interest

Dr. Weber has consulted for and accepted honoraria from Bristol Myers Squibb.

## Authors’ contributions

WSW conducted the microarray studies, flow cytometry studies, participated in the statistical analysis, data interpretation and drafted the manuscript. DHY supervised the statistical analysis, data interpretation and contributed to manuscript preparation. AAS collected clinic information of the patients. BY participated the microarray studies. MH participated discussion of the study. DM processed the study samples. YHZ performed the data analysis of microarray study, interpreted microarray data, and contributed to the manuscript preparation. XHZ conducted all the flow cytometry biomarkers analysis. JSW conceived the study, participated in the design and coordination, drafted the manuscript and supervised the entire study. All authors read and approved the final manuscript.

## Supplementary Material

Additional file 1 **Supplementary Tables and Figures. Table S1a**: Demographic data for the 12 patients for which microarray data are shown in Table 2. **Table S1b**: Demographic data for the 25 patients for which flow cytometry data are shown in Table 3a. **Table S1c**: Characteristics of 37 patients for which flow cytometry data are shown in Table 3b and Table 4. **Figure S1**: Gating strategy and representative biomarkers in the study. Additional file 1: **Figure S2**: Box plot illustrates the % of EOMES ^+^ Ki67 ^+^ CD8^+^ T cells in normal donors, relapsed and NED melanoma patients in the trial. Whiskers in box plots indicate maximum and minimum values measured. Cross indicates the mean, while line indicates the median. P-values in the graph are from Wilcoxon rank-sum test. Overall p-value = 0.038 for comparing the three groups from Kruskal-Wallis test. Click here for file
