# Supplementary material for: Biomarkers on melanoma patient T Cells associated with ipilimumab treatment
Source: J Transl Med. 2012 Jul 12;10:146. doi: 10.1186/1479-5876-10-146 (PMC3527361; doi:10.1186/1479-5876-10-146)
Supplement: Additional file 1 — Supplementary Tables and Figures. Table S1a: Demographic data for the 12 patients for which microarray data are shown in Table 2. Table S1b: Demographic data for the 25 patients for which flow cytometry data are shown in Table 3a. Table S1c: Characteristics of 37 patients for which flow cytometry data are shown in Table 3b and Table 4. Figure S1: Gating strategy and representative biomarkers in the study. Additional file 1: Figure S2: Box plot illustrates the % of EOMES + Ki67 + CD8+ T cells in normal donors, relapsed and NED melanoma patients in the trial. Whiskers in box plots indicate maximum and minimum values measured. Cross indicates the mean, while line indicates the median. P-values in the graph are from Wilcoxon rank-sum test. Overall p-value = 0.038 for comparing the three groups from Kruskal-Wallis test. [file 1479-5876-10-146-S1.pdf]

Legend for supplementary

Supplementary Table 1a: Demographic data for the 12 patients for which microarray data are shown in table 2.

Supplementary Table 1b: Demographic data for the 25 patients for which flow cytometry data are shown in table 3a.

Supplementary Table 1c: Characteristics of 37 patients for which flow cytometry data are shown in table 3b and table 4a and 4b.

Supplementary Figure 1: Gating strategy and representative biomarkers in the study.

Supplementary Figure 2:  Box plot illustrates the % of EOMES+Ki67+CD8+ T cells in normal donors, relapsed and NED melanoma patients in the trial. Whiskers in box plots indicate maximum and minimum values measured. Cross indicates the mean, while line indicates the median. P-values in the graph are from Wilcoxon rank-sum test. Overall p-value=0.038 for comparing the three groups from Kruskal-Wallis test.

Supplementary Table 1a. Patient Demographics for the Microarray cohort (n=12)

| Variable | Level | n (%) |
|---|---|---|
| gender | F | 4 ( 33.3) |
| | M | 8 ( 66.7) |
| stage | III | 4 (33.3) |
| | IV | 8 (66.7) |
| HLA A2 | A2+ | 7 (58.3) |
| | A2- | 5 (41.7) |
| dosage | 10 mg/kg | 12 (100) |
| | 3 mg/kg | 0 (0) |
| irAE | N | 7 (58.3) |
| | Y | 5 (41.7) |
| Outcome | NED | 12 (100) |
| | Relapse | 0 (0) |

Supplementary Table 1b. Demographics of patients with 3-month measurements (n=25)

| Variable | Level | n (%) |
|---|---|---|
| gender | F | 9 ( 36.0) |
| | M | 16 ( 64.0) |
| stage | III | 10 ( 40.0) |
| | IV | 15 ( 60.0) |
| HLA A2 | A2+ | 18 ( 72.0) |
| | A2- | 7 ( 28.0) |
| dosage | 10 mg/kg | 16 ( 64.0) |
| | 3 mg/kg | 9 ( 36.0) |
| irAE | N | 15 ( 60.0) |
| | Y | 10 ( 40.0) |
| Outcome | NED | 17 ( 68.0) |
| | Relapse | 8 ( 32.0) |

Supplementary Table 1c. Demographics of patients with 6-month measurements (n=37)

| Variable | Level | n (%) |
|---|---|---|
| gender | F | 12 ( 32.4) |
| | M | 25 ( 67.6) |
| stage | III | 17 ( 45.9) |
| | IV | 20 ( 54.1) |
| HLA A2 | A2+ | 20 ( 54.1) |
| | A2- | 17 ( 45.9) |
| dosage | 10 mg/kg | 31 ( 83.8) |
| | 3 mg/kg | 6 ( 16.2) |
| irAE | N | 23 ( 62.2) |
| | Y | 14 ( 37.8) |
| Outcome | NED | 27 ( 73.0) |
| | Relapse | 10 ( 27.0) |

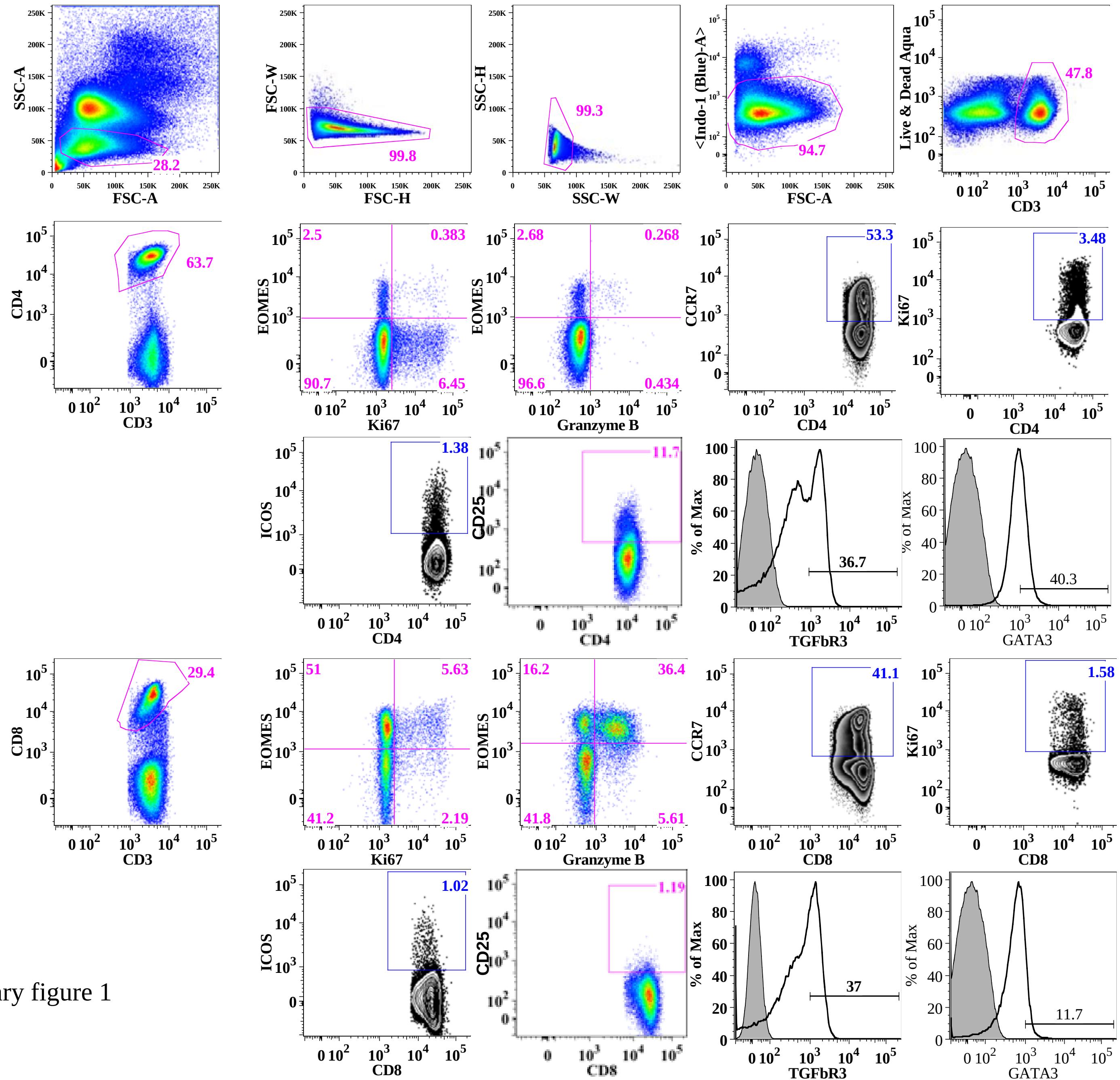


Supplementary figure 1

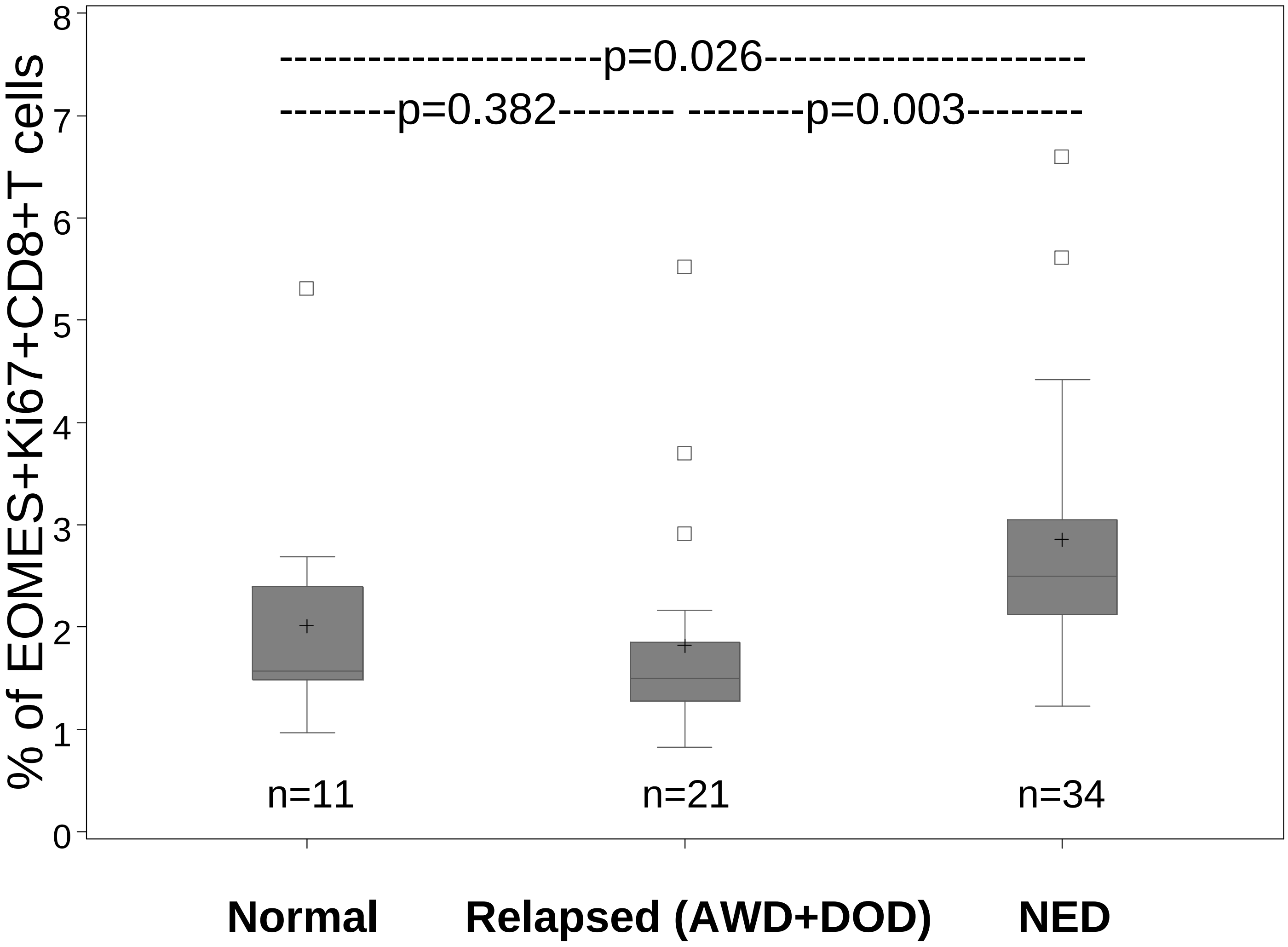


Supplementary figure 2
